# Teaching on the Frontlines: A Narrative Inquiry of Teachers' Experiences With School‐Based Trauma

**DOI:** 10.1111/josh.70193

**Published:** 2026-07-16

**Authors:** Jennifer L. Caruso, Sonali Rajan

**Affiliations:** ^1^ Department of Health Behavior and Health Equity, School of Public Health University of Michigan Ann Arbor Michigan USA; ^2^ Department of Health Studies and Applied Educational Psychology, Teachers College Columbia University New York New York USA

**Keywords:** educator well‐being, narrative inquiry, secondary traumatic stress, teacher trauma, Whole School, Whole Community, Whole Child (WSCC)

## Abstract

**Background:**

This study is grounded in the Whole School, Whole Community, Whole Child (WSCC) model, which highlights the interconnectedness of student and staff well‐being in promoting healthy, safe, and supportive school environments. It explores how school‐based trauma influences teachers' well‐being and professional identity.

**Methods:**

Utilizing a qualitative, narrative inquiry approach, four K‐12 teachers in New York City Title I charter schools participated in three semi‐structured interviews and one school‐based observation. Data were analyzed thematically and represented through a narrative documentary script.

**Results:**

Findings are organized around four narrative threads: (1) trauma's impact on teacher well‐being, (2) the emotional burden of unresolved student trauma, (3) post–COVID‐19 disruption to school climate, and (4) purpose and teacher leadership as sources of resilience. Participants described trauma exposure as cumulative over time and influential in shaping their professional identities and leadership trajectories.

**Implications for School Health Policy, Practice, and Equity:**

Trauma‐informed professional learning, accessible mental health resources for staff, and opportunities for teacher leadership are essential in building and maintaining safe, equitable, and resilient schools.

**Conclusions:**

Centering teacher narratives reveals how direct and secondary trauma affect educators' health and capacity to sustain supportive learning environments for the students they serve.

The Whole School, Whole Community, Whole Child (WSCC) model emphasizes that school environments must support the health and well‐being of students and staff to promote academic success and positive developmental outcomes [1]. Classroom environments shaped by teachers, both physically and emotionally, influence students' learning, development, and sense of safety. Teachers also build rapport, provide support, and foster a sense of belonging among students [2, 3, 4]. A healthy school climate includes not only protection from violence and harm but also strong, supportive educator‐student relationships [1, p. 733].

Within this context, teachers play a critical role in student health as instructional leaders, sources of emotional support, and behavioral guides, while maintaining connections with families and communities [5, 6, 7]. Teachers are often the first to recognize student trauma or distress, report abuse, and provide stability during crises [8, 9, 10, 11]. However, when teachers themselves experience trauma, their ability to fulfill these roles may be compromised, with consequences for their own well‐being and the students in their care [12, 13, 14].

Workplace trauma, or occupational trauma, is associated with occupational stress and negative mental health outcomes [15]. In education, this has become an increasing concern as teachers are frequently exposed to distressing experiences. The current study operationalizes this exposure as both direct and indirect trauma experienced by teachers in K‐12 school settings.

*Direct* trauma exposure occurs when teachers witness or are victims of traumatic events at school, such as violence or natural disasters [9, 12, 16, 17]. These incidents disrupt school safety and climate and can elicit emotional responses consistent with post‐traumatic stress disorder (PTSD) [9, 15, 18]. Although trauma's effects on students are well documented [19, 20, 21, 22], less attention has been given to its impact on educators, who are often expected to provide stability and care in the aftermath [23]. *Indirect* trauma exposure occurs when teachers work closely with students who have experienced trauma, leading to secondary traumatic stress (STS). STS arises when educators empathize with students' trauma but lack the ability to intervene and can produce symptoms similar to PTSD [24, 25, 26].

Recent national data from the 2023 Youth Risk Behavior Survey indicate that 76.1% of US high school students have experienced at least one adverse childhood experience (ACE) [27], emphasizing the high prevalence of student trauma and the risk of both direct and secondary trauma among educators. Despite increased attention to student trauma, critical gaps remain in understanding how teachers process school‐based trauma and how these experiences shape their mental health and teaching practices. Supporting teacher well‐being is essential not only for educators but also for student health and success [28, 29].

This study draws on narrative inquiry methodology to examine the lived experiences of teachers who have encountered workplace trauma. Through in‐depth data collection with four K‐12 teachers in public charter schools in New York City, this novel study explores: (1) how teachers describe the impact of school‐based trauma on their mental and physical health, and (2) how these experiences shape their professional identity.

## 1 Methods

### 1.1 Narrative Inquiry

Narrative inquiry was selected to center the lived experiences of teachers exposed to trauma in school settings. This methodology is particularly well‐suited for work with teachers, as it aligns with how educators already make sense of their practice [30]. Grounded in the study of experience as story, narrative inquiry examines how individuals interpret and make meaning of events and relationships that shape their lives [31]. Rather than cataloging trauma symptoms through quantitative methods, this approach enables exploration of how school‐based trauma shapes teachers' identities, relationships, and emotional well‐being over time [32].

### 1.2 Positionality

**FIGURE 1:**
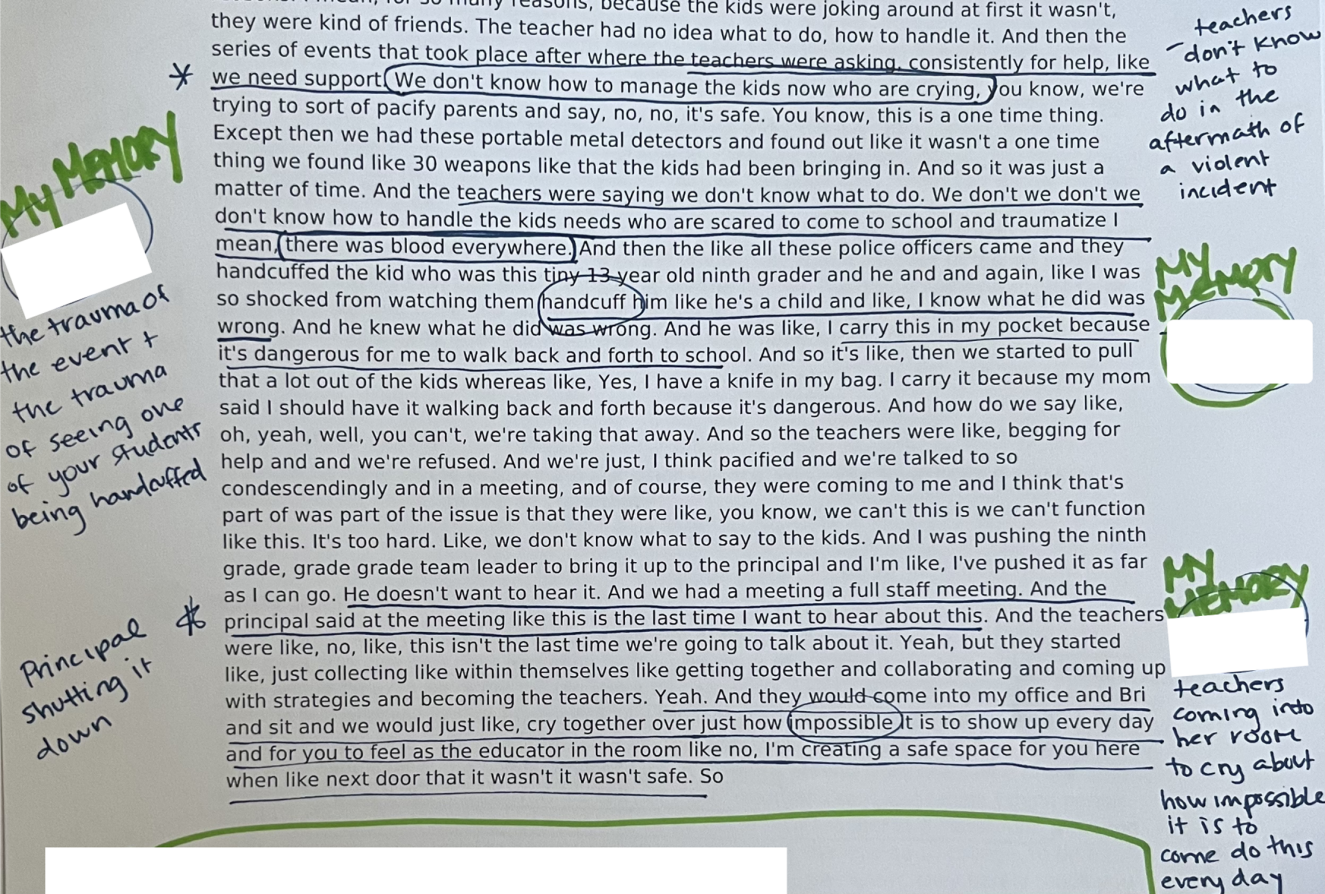
Example of reflexive practice in a narrative memo. Excerpt from a narrative memo illustrating the PI's reflexive practice. This memo was written directly on a transcription document following an interview with “Summer,” whose story prompted the researcher's recollection of a personal school‐based trauma from their former teaching career. These personal reflections are shown in green text labeled “My Memory.”

Clandinin and Connelly [31] describe narrative inquiry as engaging researchers and participants “in the midst” of experience, recognizing the ongoing and contextual nature of their lives. Reflexivity was used to make the researcher's role and influence transparent. The PI, a former teacher in a Title I charter school, drew on their teaching background throughout the study. This shared experience likely facilitated rapport and disclosure but may also have shaped assumptions about shared understanding. This influence was made explicit through narrative memo writing (Figure 1) and a self‐narrative examining the PI's own experiences with systemic pressures and the emotional demands experienced during their own teaching career [33]. These memos were kept separate from participant transcripts and used to identify assumptions, emotional responses, and potential biases rather than shape participant narratives. Participant voices remained central, with the researcher's perspective made visible without overshadowing participant narratives.

One participant had previously worked with the PI as a teacher approximately ten years before the study. This relationship was collegial, with no supervisory or evaluative authority, and did not involve the school described in the narrative. No ongoing professional relationship existed at the time of recruitment or data collection. No prior relationships existed with the remaining participants.

### 1.3 Participants

Narrative inquiry emphasizes relational, interactive engagement between researchers and participants [34, 35]. Consistent with this approach, participants were actively involved throughout the research process, including ongoing consent, opportunities to clarify or decline questions, and the ability to revise their narratives during member checking [31, 35, 36, 37]. Final narrative representations were developed through an iterative review process to ensure accuracy, respectful portrayal, and alignment with participants' intentions.

Because narrative inquiry emphasizes understanding individuals' unique experiences rather than striving for generalizability [38], this study emphasized depth over sample size, developing rich accounts of each participant's lived experience. Multiple interviews conducted across the school year, in‐school observations, artifact sharing, and iterative member checking produced temporally grounded, contextually resonant narratives sufficient to address the research questions.

Four K‐12 teachers from Title I charter schools in New York City participated. Their self‐selected pseudonyms are Summer, Dawn, Alex, and Olivia. At the time of data collection, Olivia worked at an elementary school in Queens, Alex at a middle school in Queens, Summer at a high school in the Bronx, and Dawn at a high school in Brooklyn. Although diverse in age, gender, and grade level, all had 10–15 years of teaching experience and reported at least one school‐based traumatic event since March 2020.

### 1.4 Recruitment

Participants were recruited through a digital flyer shared on the PI's professional social media accounts, including Instagram (@teachwellnyc) and LinkedIn. The Instagram post was also boosted as a targeted advertisement for New York City teachers. Participation was voluntary, uncompensated, and independent of any supervisory or evaluative relationship with the PI. While 19 individuals responded, most did not meet the inclusion criteria. Eligible participants were current teachers in New York City Title I charter schools who had experienced at least one school‐based traumatic event since March 2020 and demonstrated a strong commitment to collaboration.

## 2 Instrumentation

Teacher experiences with school‐based trauma were defined as exposure to potentially traumatic events occurring within the school environment during the workday. Direct trauma exposure was informed by categories from the Life Events Checklist for the DSM‐5 (LEC‐5) [39], including events teachers witnessed or experienced at school. This definition also included indirect exposure through STS. These experiences were explored through semi‐structured interviews conducted with each teacher at five points (meetings) across the school year to capture how experiences and narratives evolved over time (Table 1). Semi‐structured interview questions were used (Appendix A). Interviews were conducted across multiple modalities, including Zoom, in‐person “in the midst” [32] school observations, artifact sharing, and email correspondence, to support a richer understanding of each participant's experience.

**TABLE 1:** Structure and intent of PI/participant meetings.

First meeting: Zoom interview #1	Second meeting: In‐person school observation	Third meeting: Zoom interview #2	Fourth meeting: Member checking #1	Fifth “meeting” (via email): Member checking #2
Continued rapport building Participant provided with operational definitions of trauma. Participant invited to share experiences as stories. Q&A.	PI spent time “in the midst” [31] with participants as they moved through their school day, paying close attention to the narrative inquiry constructs of place and embodiment. Data collection consisted of both observation and interview questions.	Participant shared an artifact representing their professional identity as a teacher. Participant invited to reflect on any additional details or insights regarding the shared experience. Discussed available trauma support and identified gaps.	PI shared the narrative with the participant to confirm accuracy. The PI and participant reflected on the process of co‐creating data as research participants PI made edits to the narrative based on the participant's feedback.	PI shared the final narrative documentary script with each participant via email to confirm accuracy. PI made edits to the narrative documentary based on the participant's feedback.

## 3 Procedures

### 3.1 Interview Setting

Two of the three interviews for each participant were conducted via Zoom, alongside a full‐day, in‐person school visit, where the PI observed teachers “in the midst” of their school day [31]. To develop a holistic understanding of participants' experiences at school, the researcher observed teachers across multiple settings, including classrooms, hallways, and prep periods. This immersive approach reflects narrative inquiry's emphasis on “place,” [30] attending to the physical, social, and contextual environments that shape participants' experiences.

### 3.2 Interview Recording and Transcription

All interviews were audio‐recorded with participants' consent and transcribed verbatim. Transcripts served as the primary data source, supplemented by narrative memos and contextual observations collected during school visits.

### 3.3 Narrative Memoing

Narrative memoing was used to document how participants' experiences were interpreted across the study as meanings developed over time [40, 41]. In this study, memos included annotations on transcripts (Figure 1) and detailed field notes from in‐person school observations, capturing the PI's in‐the‐moment reflections during multiple levels of data collection.

## 4 Data Analysis

**FIGURE 2:**
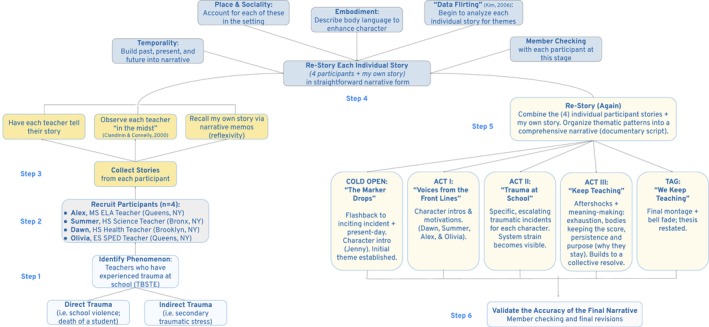
Six‐step data collection and analysis procedure.

Data were collected and analyzed using a six‐step process (Figure 2) grounded in narrative inquiry methodology [31, 42, 43]. Rather than applying a rigid coding structure, this study followed Kim's [44] “data flirting” approach, which emphasizes open, iterative engagement with the data as patterns and meanings emerge. This process involved printing and physically organizing transcripts, memos, and field notes to group and reshape emerging storylines.

The PI reconstructed four individual narratives using Bruner's [45] meaning‐making framework, attending to temporality, sociality, place, and embodiment [31]. These narratives were then re‐storied into one cohesive script guided by Polkinghorne's [43, 46] model of narrative configuration. Member checking ensured that participants approved their narrative representations. A thematic matrix was used to identify shared patterns and connect quotes across participants.

Trustworthiness was supported through prolonged engagement across interviews, a comprehensive in‐school observation, artifact sharing, and iterative member checking. Reflexive memo writing documented analytic decisions alongside the researcher's interpretive process. Multiple data sources (interviews, observations, artifacts, memos) supported narrative reconstruction and interpretation.

### 4.1 Researcher Role in Interpretation

The researcher's background as a former teacher informed interpretation, and reflexive memoing supported transparency. Findings should therefore be understood as contextually situated, co‐constructed narratives.

### 4.2 Presentation of Findings Through Narrative Documentary

Findings were presented in a narrative documentary script [47] to preserve participants' voices and convey the emotional and temporal complexity of the trauma experienced. The structure drew on documentary and “mockumentary” conventions (e.g., *The Office*, *Parks and Recreation*, *Abbott Elementary*) and adapted them to reflect the seriousness of the subject matter. Selected narrative documentary scripts [48, 49] informed decisions related to formatting, pacing, and tone in shaping how participants' stories were represented.

Storytelling techniques such as flashbacks, voiceovers, and reflective interviews were used to illustrate how participants revisited and reframed past events [31, 45, 50]. Stories were constructed with attention to temporality, social context, and identity to reflect how experiences were shaped and understood over time [51]. “Talking Head” interviews [52] were used to foreground participants' retrospective sensemaking, capturing how they interpreted and assigned meaning to their experiences as they looked back on them. Parallel “Talking Head” segments from the PI were included to make the researcher's reflexive sensemaking visible, capturing how the PI interpreted and revisited their own experiences in relation to participants' narratives.

The documentary format served as a representational structure rather than fictional storytelling. Participant dialogue was drawn directly from transcripts and presented verbatim, including filler words and natural speech patterns. Descriptions of school settings were based on participant accounts and the PI's in‐school observations. Narrative reconstruction involved selecting and sequencing events to reflect participants' narrative arcs, guided by temporal order and narrative significance. No dialogue, events, or characters were fictionalized. Participants reviewed individual narratives and the final script during member checking to ensure accuracy.

Although this format may raise concerns about trivializing trauma, it was intentionally selected to center each teacher's voice and represent the complexity of participants' experiences within school contexts, consistent with narrative inquiry.

### 4.3 Ethical Considerations

This study was approved by the Teachers College Institutional Review Board. Participants provided informed consent and were informed of study procedures, risks and benefits, confidentiality protections, and their right to withdraw at any time. Interviews were conducted using a trauma‐informed approach. Participants could pause, skip questions, or stop at any time and were informed of the researcher's mandatory reporting responsibilities in cases of current risk. Interviews were transcribed from audio recordings; audio files were destroyed after transcripts were received and verified. All data were stored on password‐protected platforms accessible only to the researcher.

## 5 Results

Study findings emerged through narrative reconstruction and are presented in a documentary‐style script examining participants' experiences across time, relationships, and school contexts. Findings are organized around four interrelated themes; however, these themes are understood as narrative threads that run across participants' stories, reflecting patterns in how they made sense of their experiences. These threads are illustrated through excerpts from participants' words within the documentary script.

### 5.1 “It Changes You”: School‐Based Trauma and Its Impact on Teacher Well‐Being

Across participants' stories, school‐based trauma had lasting effects on physical and emotional well‐being. Teachers described how repeated exposure accumulated over time, affecting sleep, health, and emotional functioning. Participants reported anxiety, fatigue, sleep disruption, and physical illness. As Summer shared, “At the end of every year, I break down… my body just can't take it anymore.” Olivia similarly noted, “That story… it still keeps me up at night.”

Participants also described emotional exhaustion, anxiety, and numbness. Dawn reflected, “I'm guarded now. There's a wall up that wasn't there before.” Olivia explained, “These stories… become a part of you… we carry their stories. And that weight… it changes you.” Across narratives, trauma was experienced as ongoing and cumulative, shaping teachers' well‐being, relationships, and perception of their work.

### 5.2 “We Did Everything We Could”: Limits of Influence and Unresolved Trauma

Teachers described the emotional strain of caring deeply for students while having a limited ability to change outcomes. Many invested significant time and emotional energy, yet experienced unresolved grief when students' situations did not improve. Olivia recalled, “We did everything we could… but after the abortion, [the student] just ran. We never saw her again.”

Teachers also described schools moving too quickly past traumatic incidents, leaving no space for processing. Summer reflected on witnessing a violent incident between students: “We were told to just… stop talking about it. Move on, like it never happened… But how do you move on from something like that?” These experiences were particularly difficult due to the lack of closure, as teachers were often left without resolution yet continued to carry concern for students over time.

### 5.3 “The [COVID‐19] Pandemic Changed Everything”: Lasting Impact on School Communities

Participants described the COVID‐19 pandemic as a turning point that reshaped school communities, student needs, and available supports. Teachers reported pressure to return to “normal” without space to process collective trauma. As Alex shared, “[post‐COVID] the kids came back… and we were just expected to act like everything was normal.”

Participants also described breakdowns in the school community and reduced administrative support. Alex noted, “The pandemic… it changed everything. The entire school community was lost. Before COVID, there were reports, follow‐ups, and genuine concern. After COVID, it was just, ‘File this report.’” Across narratives, the pandemic marked a shift toward greater student needs alongside fewer supports, leaving teachers feeling more responsible yet less supported.

### 5.4 “They Need Us”: Purpose and Leadership as Staying in the Profession

A notable pattern across all four narratives was that each teacher stepped into a leadership role following their experiences with school‐based trauma. Rather than leaving the profession, participants described using these experiences to take on leadership roles and advocate for change. Leadership served as a strategy to make meaning of their experiences while also supporting colleagues facing similar challenges and continuing to be present for their students. As Alex reflected, “These moments, as painful as they are, embolden me to keep doing this work. They need us.”

Dawn described designing and facilitating boundary‐setting workshops for staff, stating, “I feel like I'm doing what I'm supposed to be doing here.” Olivia described how her leadership role allowed her to support teachers in ways she had needed earlier in her career: “Our school is a community. They come to me… my teachers, my staff… just to check in.” Across narratives, leadership served to transform difficult experiences into a sense of purpose and motivation to remain in the profession while working to improve conditions for others.

## 6 Discussion

This study examined how teachers made sense of school‐based trauma and how these experiences shaped their personal and professional lives. Across narratives, trauma was not described as isolated incidents but as cumulative experiences influencing teachers' responsibilities, relationships, and decisions to remain in the profession. Many experiences functioned as turning points, shaping professional identity and, for all participants, contributing to movement into leadership roles. These findings suggest that school‐based trauma affects teacher well‐being, professional identity, career trajectories, and sense of purpose.

First, this study contributes to research on school‐based trauma by centering teacher perspectives, which are often overlooked in school health and safety literature. Consistent with prior work on STS [17, 53, 54, 55] participants described exhaustion, anxiety, sleep disruption, and emotional withdrawal. Their narratives also highlight how trauma is cumulative and integrated into teachers' lived experiences, shaping how they understand their roles and relationships, aligning with evidence linking repeated trauma exposure to mental health outcomes [56].

Second, findings highlight the emotional toll of caring for students while having a limited ability to influence outcomes. Participants described grief, helplessness, and frustration when students' situations did not improve, particularly when schools moved quickly past traumatic events without space for processing. These accounts align with prior research linking insufficient support to burnout and helplessness [55]. They may also reflect elements of moral injury, in which individuals experience distress when unable to act in accordance with their values [56], as well as institutional betrayal, when organizations fail to provide adequate support following trauma [57].

Third, participants described the COVID‐19 pandemic as a turning point that reshaped school climate, student needs, and available supports. Teachers reported pressure to return to “normal” without processing collective trauma, alongside reduced communication and support. These findings align with research on the pandemic's effects on school climate and connectedness [58].

Finally, leadership emerged as a key mechanism through which teachers made meaning of their experiences. All participants described stepping into leadership roles following trauma exposure, using these roles to support others and advocate for change. This aligns with research suggesting that purpose and ethical commitment can support resilience and job satisfaction [59], and may reflect post‐traumatic growth [60].

### 6.1 Implications for School Health Policy, Practice, and Equity

These findings highlight the importance of prioritizing teacher well‐being within comprehensive school health frameworks. The Whole School, Whole Community, Whole Child (WSCC) model and trauma‐informed approaches (e.g., HEARTS) emphasize that school health depends on supportive environments for both students and staff [61]. Participants described trauma exposure as cumulative and often unresolved, suggesting that individual‐level strategies alone are insufficient. Instead, schools require organizational structures that acknowledge and respond to school‐based trauma.

Teachers function as key relational supports for students, often serving as a “secure base” or “safe haven.” [62, 63] If teachers are expected to fulfill this role, they must also be supported following traumatic events. When teachers lack adequate support, their capacity to provide students with stability is undermined, making teacher well‐being itself a matter of student health.

Teachers also play a critical role in preventing and responding to student trauma. When teachers experience trauma themselves, their ability to maintain safe and supportive environments may be affected [64]. School health practices should therefore include trauma‐informed professional development, access to mental health resources, and structured opportunities for processing traumatic events. Schools may also benefit from creating pathways for teacher leadership and participation in decision‐making as strategies for retention and system improvement.

Equity remains central to these efforts. Conducted in Title I schools, this study reflects contexts shaped by structural inequities, where teachers may face heightened exposure to trauma and its disproportionate impacts, including those intensified by the COVID‐19 pandemic [65, 66, 67]. These findings suggest that trauma‐informed approaches must extend beyond student support to address the structural and organizational conditions shaping teacher experiences, particularly in historically marginalized communities.

### 6.2 Limitations

This study has several limitations that should be considered when interpreting its findings. Narrative inquiry prioritizes depth over generalizability; therefore, the small sample size does not reflect the full diversity of educators in Title I charter schools or other school settings [31, 68]. All participants worked in urban charter schools in New York City, which may differ from public, private, or rural schools in terms of context, resource availability, student needs, and organizational supports.

Recruitment through the PI's public‐facing professional social media accounts may have attracted teachers already engaged in professional networks or more inclined toward reflecting on their teaching practice. As a result, the sample may not represent educators who are less connected on social media or less comfortable sharing personal narratives.

Additionally, teachers' experiences were shaped in part by the ongoing impact of the COVID‐19 pandemic, which may have influenced how trauma was perceived and processed during this period. The long‐term effects of experiencing trauma at school may also continue to develop and accumulate beyond the data collection period.

An additional limitation is that all participants were current teachers at the time of the study and had moved into leadership roles within their schools. As a result, the findings reflect the experiences of teachers who remained in the profession and transitioned into leadership following school‐based trauma. Leadership may not function as a source of resilience for all teachers, as increased responsibility, emotional labor, and institutional pressures may contribute to additional stress or burnout. Teachers who left the profession or chose not to pursue leadership roles may have had different experiences and may not have found leadership or continued teaching to be a source of resilience. Future research should examine diverse professional trajectories following school‐based trauma, including teachers who remain in the classroom, transition to other roles, or leave the profession.

Finally, because this study used a narrative inquiry approach, findings were developed through narrative reconstruction and interpretation, involving the selection, sequencing, and representation of participants' experiences in narrative form. Although this process was conducted collaboratively through member checking and ongoing dialogue, narrative representation is inherently interpretive and shaped by the researcher's perspective.

## 7 Conclusions

This study contributes to the growing body of school health research by illustrating how teachers make meaning of school‐based traumatic experiences over time and how these experiences shape their well‐being, professional identities, and career trajectories. Findings demonstrate that school‐based trauma affects not only students but also the educators who support them, highlighting the importance of including teachers within school health and safety frameworks.

Methodologically, this study demonstrates the value of narrative inquiry in capturing teachers' lived experiences of trauma within school contexts. By presenting findings through a documentary‐style script, this study offers an accessible and engaging format designed to resonate with educators, school leaders, and practitioners, extending the reach of research beyond traditional academic audiences.

These findings have implications for school health policy and practice. Supporting teacher well‐being should be a core component of trauma‐informed and whole‐school approaches. Schools and districts should consider policies that provide access to mental health resources, opportunities for reflection and peer support, and pathways for teacher leadership and advocacy related to school climate and trauma‐informed practices.

Although this study focused on a small sample of teachers in Title I charter schools in New York City, it highlights several directions for future research. Future studies should examine how school context and organizational conditions shape teachers' experiences of trauma and explore diverse professional trajectories, including teachers who remain in the classroom, transition to different roles, or leave the profession. Longitudinal research may further illuminate how trauma narratives and professional identities evolve over time.

At its core, this study is about the stories teachers carry and the meaning they make from difficult experiences in their professional lives. The teachers in this study shared deeply personal and often painful experiences, highlighting the emotional and relational labor that is often invisible in discussions of school health. Their narratives remind us that teachers are not only educators, but also witnesses, caregivers, advocates, and leaders working within complex and often challenging school environments. Listening to and learning from teachers' stories is essential not only for supporting teacher well‐being but also for creating healthier, safer, and more supportive school communities for both teachers and the students they serve.

## Ethics Statement

This study was approved by the Teachers College, Columbia University Institutional Review Board (Protocol #24‐136). All participants provided informed consent prior to participation.

## Conflicts of Interest

The authors declare no conflicts of interest.

## Data Availability

The data that support the findings of this study are available from the corresponding author upon reasonable request.

## Appendix A Semi‐Structured Interview Questions

### Interview #1: 45–60 min

Please describe/introduce yourself.

- What pseudonym would you like to use?
- What pronouns do you use?
- Do you identify with a specific gender?
- Do you identify with a specific race?
- Aside from teaching, what should I know about you? What are your interests and hobbies?

Please describe your teaching experience.

- What grade and subject do you teach?
- How long have you been working in education?
- How long have you been working in your current school?
- Why did you choose to become a teacher?
- How would you describe your teaching style?

[Describe narrative inquiry, their role as “collaborators,” and how storytelling will be used in this study.] I'm hoping to get to know more about who you are as a person and to practice answering questions in narrative form.

- Can you share a story about something that happened to you in the past week that has nothing to do with teaching?
- How did it feel to share this experience in the form of a story?

[Share the definitions that will be used in this study.] Do you have any questions about any of these definitions?

- Which, if any, of these definitions stood out to you or resonated with you?

Please share your story about a school‐based traumatic experience.

- Can you describe any significant experiences or instances of trauma you've encountered while working in a Title 1 charter school in NYC since March 2020?

Possible follow‐up questions:

- When reflecting on this particular incident, could you describe the emotions or thoughts you experienced during that time?
- How have these experiences impacted your overall well‐being and day‐to‐day functioning as a teacher?
- How do you cope with or manage stress and emotional challenges resulting from these traumatic experiences while maintaining your professional responsibilities?

I'd like to ask you some questions about the impact of this experience on your life

- Could you describe how these school‐based traumatic experiences have manifested in your daily life, either mentally or physically?
- Have you sought any formal or informal support to address the mental or physical impacts of these experiences?

Possible follow‐up questions:

- Could you talk about any support networks or resources, both within or outside the school, that were particularly helpful or supportive to you during these challenging times?
- How did the support received, or lack thereof, impact your ability to process and move forward from these traumatic events?

Now I'd like to ask you a few questions about how this experience affected your classroom:

- Have you noticed any changes in your motivation or passion for teaching due to these experiences?
- Have you observed any changes in the overall classroom dynamics or student behavior following these traumatic incidents?

Possible follow‐up questions:

- Can you recall any specific instances where your relationship with a student was affected by a traumatic event?
- Can you elaborate on how this specific traumatic event affected your teaching practices or your interactions with colleagues and students?

At our next interview, I will invite you to share an artifact (art, music, image) that helps you tell your story about the event.

### Interview #2: 45–60 min

Please reflect on our previous interview.

- How did it feel to share your story about school‐based trauma with someone?

Possible follow‐up questions:

- Do you believe that sharing your story has impacted your own understanding or processing of those experiences?
- Were there any details about the experience that you didn't share at the first interview that you would like to share now?

I'd like to invite you to share an artifact that represents your experiences with the traumatic event we've been talking about:

- Can you describe the significance or symbolism behind the artifact you chose to represent your story? How does it relate to the narrative you tell yourself about that event?
- What specific elements or details within the artifact resonate most strongly with your traumatic experience?

Possible follow‐up questions:

- How does the process of engaging with this particular form of expression (art, music, image) influence your understanding or processing of the narrative?
- How does engaging with this artifact contribute to your ongoing narrative or understanding of traumatic events in your life as a teacher?

I'd like to revisit our conversation from our last interview regarding the support you received, or lack thereof, following the traumatic event we've been discussing:

- How did the school community or administration respond to the traumatic incident?
- Did you feel adequately supported by your school community in the aftermath?
- How did the support received, or lack thereof, impact your ability to process and move forward from these traumatic events?
- Were there any professional development opportunities or training sessions provided by the school that assisted you in handling similar situations in the future?

Possible follow‐up questions:

- Are there any specific areas or aspects you believe should receive more attention or resources to better support teachers in Title 1 charter schools dealing with trauma?
- How do you think additional resources or services could address the gaps you've identified in supporting teachers through traumatic experiences?

I'm hoping we can use our remaining time together to reflect on the process of co‐constructing a narrative.

- What are the key takeaways or insights you've gained from participating in this process of sharing and validating your story?
- To what extent did you feel involved and empowered in shaping the narrative/story about your traumatic experience? Were there moments when you felt your voice was particularly influential?
- What insights or learnings have you gained from engaging in this collaborative process to articulate your traumatic experience?

Possible follow‐up questions:

- Reflecting on the collaborative effort to construct this narrative/story, what value do you believe this process adds to your own understanding or representation of the traumatic experience?
- How might this collaborative narrative‐building process positively impact others' understanding or empathy toward educators facing challenges with school‐based trauma?

### Interview #3: 45–60 min

This is a member checking interview to validate and ensure the accuracy of the narrative. During this session, the completed narrative will be shared with each participant for their review.

Please reflect on your perspective of the narrative that has been created to tell your story

- As you've had a chance to review the narrative that encapsulates your traumatic experience, could you share your immediate thoughts or feelings upon first reading it?
- What aspects of the narrative/story resonated with your personal experiences, and were there any elements that felt inaccurate or not fully representative?

Possible follow‐up questions:

- Does the narrative/story accurately portray your voice and perspective regarding the traumatic event, or does it feel more generalized or removed from your personal experience?
- In what ways does the narrative/story accurately reflect the emotions, challenges, and nuances of your traumatic experience as you remember it?
- Are there specific details or moments within the narrative/story that you feel are misrepresented or missing crucial information?
- Are there any additional details, experiences, or emotions related to the traumatic event that you feel should be included or clarified in the narrative/story?
- Do you have any suggestions or recommendations to enhance the narrative/story and ensure it more accurately reflects your journey?
